# The Convalescent Home as a Hospital Department

**Published:** 1911-06-18

**Authors:** 


### THE CONVALESCENT HOME AS A HOSPITAL DEPARTMENT.

In glancing through the list of some 280 convalescent homes mentioned in Burdett's "Hospitals and Charities," it becomes evident that the importance of closely attaching convalescent treatment to hospital work has already forced itself on public attention. No fewer than twenty-one general and special hospitals in London alone have institutions of their own in the country or at the sea for the reception of convalescent patients. Twenty-five provincial, eighteen Scottish, and five Irish hospitals have similar accommodation, and every year the number increases, for the system of allowing hospital patients to be sent to any institution which will receive them in their convalescence is open to many objections. One hospital after another is finding that it pays better to make the convalescent home a definite hospital department, in which the treatment begun in the hospital wards can be brought to a worthy end without any shifting of responsibility.

There are several ways at present in which hospitals co-operate with convalescent homes for the benefit of their patients. An arrangement is frequently made for a certain number of beds to be set apart for hospital patients in the convalescent home attached to a particular district. This plan saves a great deal of trouble to the hospital authorities, since they have no financial or administrative responsibility in connection with the home, but at the same time there is loss of control over the conduct of the cases after they have once left the hospital. Another plan is the joint system, through which various hospitals combine for the entire use of a home. The Hospital Convalescent Home at Swanley is entirely reserved for patients from St. Thomas's, the London, Guy's, the Westminster, and St. Mary's Hospitals, and the connection between the home and the hospitals is thus far closer than is ordinarily the case.

In the first place, a considerable simplification of organisation ensues when the convalescent home is entirely dependent upon the hospital. The question of finance is no doubt a serious matter for the first year or two of the home's existence, and managers often hesitate before enlarging their obligations, fearing lest money which would otherwise be devoted to hospital needs should be diverted to a new source. When the plunge has once been taken, however, it becomes evident that there is a strong and worthy impulse in members of the public to support convalescent forms of aid, and that those homes which are managed by well-known hospitals command widespread sympathy. The same means of publicity which are adopted to make known the wants of the hospital advertise also the good work done by the home, which becomes an enterprise greatly favoured and visited by subscribers who might shrink a little from the sterner hospital associations. Moreover, it becomes an outlet for that zeal for affording Samaritan aid which is occasionally a dilemma to the hospital manager. It has been declared on high legal authority that legacies for the benefit of out-going patients may rightly be devoted to completing their restoration to health in the convalescent department, and this is certainly a far more satisfactory form of relief than presenting them with money or clothing. Again, although the management of the great majority of convalescent homes is highly creditable to the lay committees who control them, there can be no certainty that the treatment necessary for the patients is continued unless the home is under the direct control of the hospital. When hospital beds are rapidly emptied, as they are in the present day, to make way for new sufferers, it is impossible to consider the discharged patient as one whose cure is complete. Any blunder in convalescence at this early stage may, and frequently does, result in his being returned to the hospital to recommence—this time seriously handicapped—a baulked cure. That patients cannot make a satisfactory recovery in the vitiated air of the town hospital is now well understood. They must be got into a suitable environment as soon as the early critical stage of their disease has been passed, but the science and skilled service which has brought them through this first stage is wasted unless it can be continued without break of continuity through the next stage to complete recovery.

Lastly, the control of the convalescent department enables the same processes of experiment and research to be continued in the later stages of recovery which has already made the voluntary hospitals such rich benefactors to mankind. We have only need in this connection to point out the rapid advance in the treatment of convalescent consumptives which has followed the erection of the Frimley Home in connection with the Brompton Hospital for Consumption. The secrets of the prevention of disease lie somewhere hid among the processes of recuperation observable in the convalescent home. Are not these processes worthy of the best skill which the modern hospital can place at the disposal of their patients?

